# ^18^F-Fluorodeoxyglucose Positron Emission Tomography-Based Risk Score Model for Prediction of Five-Year Survival Outcome after Curative Resection of Non-Small-Cell Lung Cancer

**DOI:** 10.3390/cancers16142525

**Published:** 2024-07-12

**Authors:** Chae Hong Lim, Sang-Won Um, Hong Kwan Kim, Yong Soo Choi, Hong Ryul Pyo, Myung-Ju Ahn, Joon Young Choi

**Affiliations:** 1 Department of Nuclear Medicine, Soonchunhyang University College of Medicine, Seoul 04401, Republic of Korea; 2 Division of Pulmonary and Critical Care Medicine, Department of Medicine, Samsung Medical Center, Sungkyunkwan University School of Medicine, Seoul 03181, Republic of Korea; 3 Department of Thoracic and Cardiovascular Surgery, Samsung Medical Center, Sungkyunkwan University School of Medicine, Seoul 03181, Republic of Korea; 4 Department of Radiation Oncology, Samsung Medical Center, Sungkyunkwan University School of Medicine, Seoul 03181, Republic of Korea; 5 Division of Hematology-Oncology, Department of Medicine, Samsung Medical Center, Sungkyunkwan University School of Medicine, Seoul 03181, Republic of Korea; 6 Department of Nuclear Medicine, Samsung Medical Center, Sungkyunkwan University School of Medicine, Seoul 03181, Republic of Korea

**Keywords:** ^18^F-fluorodeoxyglucose, PET/CT, non-small-cell lung cancer, surgery, prognostic factor

## Abstract

**Simple Summary:**

The ^18^F-FDG PET parameters reflecting the intensity and distribution of glucose uptake by the tumor are associated with prognosis in non-small-cell lung cancer (NSCLC) patients. We developed and evaluated an imaging-based model utilizing these ^18^F-FDG PET-derived features for predicting the five-year survival in NSCLC patients after curative surgery. The PET-based risk score constructed using the LASSO logistic method outperformed the predictive performances of individual ^18^F-FDG PET parameters. The PET-based risk score was an independent prognostic factor for clinical variables. Additionally, it demonstrated better predictive performance when combined with clinical variables. The FDG PET-based imaging model could aid in risk stratification for personalized adjuvant treatment and follow-up management of NSCLC patients after surgery.

**Abstract:**

The aim of our retrospective study is to develop and assess an imaging-based model utilizing ^18^F-FDG PET parameters for predicting the five-year survival in non-small-cell lung cancer (NSCLC) patients after curative surgery. A total of 361 NSCLC patients who underwent curative surgery were assigned to the training set (*n* = 253) and the test set (*n* = 108). The LASSO regression model was used to construct a PET-based risk score for predicting five-year survival. A hybrid model that combined the PET-based risk score and clinical variables was developed using multivariate logistic regression analysis. The predictive performance was determined by the area under the curve (AUC). The individual features with the best predictive performances were co-occurrence_contrast (AUC = 0.675) and SUL peak (AUC = 0.671). The PET-based risk score was identified as an independent predictor after adjusting for clinical variables (OR 5.231, 95% CI 1.987–6.932; *p* = 0.009). The hybrid model, which integrated clinical variables, significantly outperformed the PET-based risk score alone in predictive accuracy (AUC = 0.771 vs. 0.696, *p* = 0.022), a finding that was consistent in the test set. The PET-based risk score, especially when integrated with clinical variables, demonstrates good predictive ability for five-year survival in NSCLC patients following curative surgery.

## 1. Introduction

Lung cancer is a leading cause of death among cancer patients worldwide, with non-small-cell lung cancer (NSCLC) comprising 85% of these cases [1,2]. Despite recent advancements in treatment modalities, therapeutic strategies based on cancer staging have limitations in providing the most effective treatments for individual patients [3,4]. Due to its reliance on identifying visible lesions, conventional staging is limited in its ability to detect microscopic cancer cells or the complex biological activities that drive tumor aggression [5]. In the era of precision medicine, a more accurate risk stratification approach could increase the efficiency of the selection of treatment options for NSCLC patients and, potentially, improve survival rates [6].

Several laboratory and pathological variables have been proposed as predictive markers for more accurate prognostic forecasting in NSCLC patients [7]. However, biomarkers derived from tissue samples are invasive, and laboratory markers obtained from blood tests reflect the secondary effects of lesions. Recently, there has been a focus on identifying non-invasive and lesion-specific prognostic imaging biomarkers employing CT-derived radiological parameters, a method referred to as radiomics [8]. Nevertheless, CT-derived structural information is limited in capturing the complex biological and functional characteristics of tumors [9].

^18^F-Fluoro-2-deoxyglucose (^18^F-FDG) positron emission tomography/computed tomography (PET/CT) has improved cancer staging and offered insights into the tumor metabolism, correlating with aggressiveness and metastatic potential [10,11]. Numerous studies have demonstrated that quantitative features derived from ^18^F-FDG PET, calculated based on the intensity and distribution of glucose uptake by the tumor, serve as valuable prognostic indicators [12,13,14,15]. Furthermore, recent studies have focused on developing imaging-based prognostic models by combining a large number of ^18^F-FDG PET-derived parameters. Although several models have also been developed for NSCLC, they often include both operable and inoperable patients [16,17]. Research targeting NSCLC patients who have received curative resection may be more clinically useful for determining the intensity of adjuvant therapy or postoperative surveillance.

Constructing predictive models for cancer prognosis using high-dimensional data with numerous features requires careful consideration. The continuous-time Cox proportional hazards model, while widely employed in survival analysis, requires the fulfillment of the proportional hazards assumption. This assumption is often violated in practice due to time-varying covariate effects and unobserved heterogeneity, which might be more prevalent in high-dimensional data [18]. A discrete-time prediction model for determining the survival status at a specific time point could offer a more suitable alternative for survival analysis with a large number of ^18^F-FDG-derived parameters [19]. We hypothesize that the integration of ^18^F-FDG PET-derived parameters with clinical variables will yield a predictive model with superior accuracy for five-year survival outcomes in NSCLC patients who have undergone curative surgery compared to models based solely on clinical data.

## 2. Materials and Methods

### 2.1. Study Population

**Figure 1:**
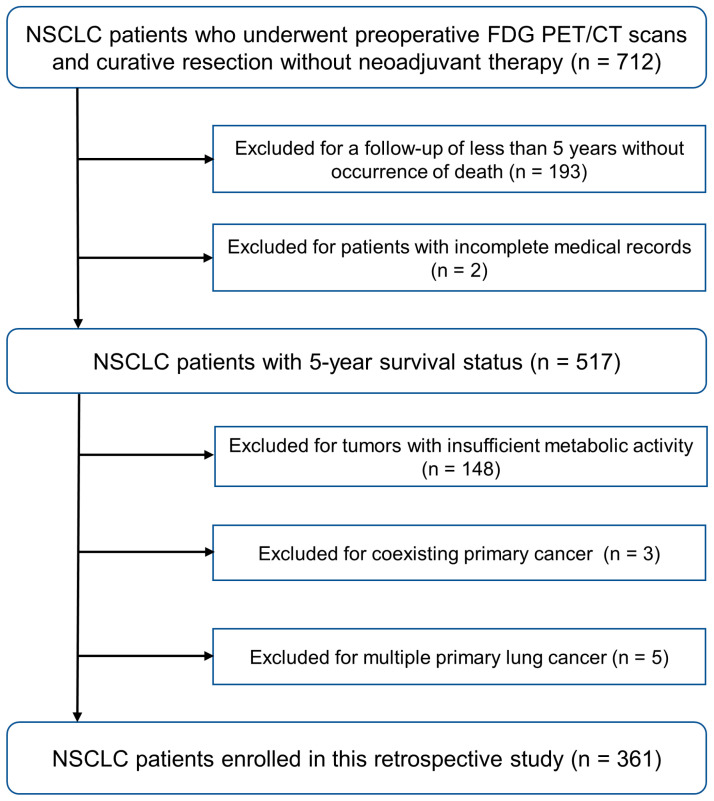
Chart of study identification and inclusion and exclusion criteria.

Our study was approved by the Institutional Review Board, which waived the need for informed consent due to its retrospective nature. Consecutive NSCLC patients who underwent curative resection between January 2016 and December 2017 were retrospectively reviewed. The inclusion criteria were as follows: (1) patients who underwent pretreatment ^18^F-FDG PET/CT scans on a GE Discovery STE scanner, (2) no neoadjuvant chemotherapy or radiotherapy, and (3) postoperative follow-up for at least five years (unless death occurred). The exclusion criteria were as follows: (1) patients with incomplete medical records, (2) tumors with insufficient metabolic activity to be delineated by an SUV cut-off of 2.5, (3) a coexisting primary cancer, and (4) cases of multiple primary lung cancers. The final study population included 361 patients. The study recruitment process is presented in Figure 1.

### 2.2. Data Collection

All clinical and pathological data were collected from electronic medical records. Clinical characteristics included age, sex, type of surgery, and adjuvant treatment history. Tumor characteristics of tumor size, histological subtype, and stage were obtained from pathologic reports. Tumor staging was conducted in accordance with the eighth edition of the American Joint Committee on Cancer (AJCC) TNM staging system. The primary clinical endpoint of this analysis was the five-year survival status, defined as either survival or death from any cause for follow-up five years after surgery.

### 2.3. FDG PET/CT Image Acquisition

All patients were instructed to fast for at least 6 h before undergoing PET/CT scans. Their blood glucose levels were maintained below 200 mg/dL at the time of the ^18^F-FDG injection. Whole-body PET and CT images were acquired 60 min post-injection of 5.0 MBq/kg ^18^F-FDG without intravenous or oral contrast, using a GE Healthcare (Milwaukee, WI, USA) Discovery STE scanner. Continuous spiral CT was performed with a 16-slice helical CT at 140 keV, with a current of 30–170 mA using AutomA mode and a section width of 3.75 mm. Subsequently, emission PET data were acquired from the head to the thigh for 2.5 min per frame in a three-dimensional mode. PET images were reconstructed using the ordered subsets expectation maximization (OSEM) algorithm with 20 subsets and 2 iterations for the Discovery STE (matrix size 128 × 128, voxel size 3.9 × 3.9 × 3.3 mm^3^), with CT data utilized for the attenuation correction.

### 2.4. FDG PET/CT Image Analysis and Feature Extraction

The volume of interest (VOI) for the lung lesion was delineated on PET images using MIM version 7.0 (MIM Software Inc., Cleveland, OH, USA). An experienced nuclear medicine physician, blinded to the clinical information except for the tumor site, identified the target tumor. Tumor segmentation was conducted using a gradient-based method (‘PET Edge’) [20]. Operators initiated contouring by dragging a cursor from the center of the lesion towards its periphery. The algorithm generated six axes, adjusting their length when a significant gradient was detected, to outline a 3D VOI around the tumor. These VOIs were exported as DICOM-RT structures and imported into the Chang-Gung Image Texture Analysis toolbox (CGITA, available at http://code.google.com/p/cgita, accessed on 1 March 2020), which is supported by MATLAB software (version 2014b; MathWorks, Inc., Natick, MA, USA), for feature extraction from the PET images [21]. The calculation of textural features involved resampling the gray level using a fixed bin width method at 0.4 SUV units, derived from 64 gray levels ranging from 0 to 25 [22]. A total of 70 ^18^F-FDG PET-derived features were calculated and categorized into several groups, as listed in Supplementary Table S1.

### 2.5. Feature Selection and Imaging-Based Risk Score Model Construction

The study cohort was randomly divided into training (70%) and test (30%) sets using the “createDataPartition” function in the “caret” package in R to ensure representative and unbiased distribution. The training set was utilized to develop a risk score model based on the ^18^F-FDG PET features to predict the five-year survival status. For selecting relevant variables to construct a predictive model, we initially evaluated the discriminative power using Receiver Operating Characteristic (ROC) curve analysis for a set of 70 features. Features exhibiting an area under the curve (AUC) of less than 0.6 were considered irrelevant and excluded from the model construction [23]. Subsequently, LASSO regression was chosen for its ability to handle high-dimensional data and prevent overfitting [24]. The optimal lambda (λ) was determined through 10-fold cross-validation, minimizing the mean cross-validation error. The “glmnet” package in R was used to perform LASSO regression. The PET-based risk score was calculated using a LASSO-based formula, created by multiplying each selected variable by its respective non-zero coefficient at the optimal λ value.

### 2.6. Statistical Analysis

All statistical analyses were conducted using the open-source software R version 4.0.2 (The R Foundation for Statistical Computing, Vienna, Austria) or MedCalc version 15.5 (MedCalc Software Ltd., Ostend, Belgium). Categorical variables were compared using the Chi-square test or Fisher’s exact test as appropriate. Continuous variables were compared between two groups using the Mann–Whitney U test. A multivariable logistic regression analysis was used to identify risk factors associated with five-year survival and to develop a hybrid model by combining the PET-based risk score with the clinical risk factors. The model performances were evaluated by calculating the AUC of the ROC curve, along with the 95% confidence interval (CI). To compare the AUC values between the models, DeLong’s test was used [25]. The optimal cut-off value for the PET-based risk score to predict binary outcomes was determined using the Youden Index from the ROC curve analysis. Calibration curves were created to assess the alignment between predicted probabilities and observed outcomes. All tests were two-sided, with statistical significance set at *p* < 0.05. The goodness of fit was determined using the Hosmer–Lemeshow test [26], where a *p* value above 0.05 indicated good calibration.

## 3. Results

### 3.1. Baseline Characteristics of Patients

The study comprised 237 male and 124 female subjects, with an average age of 63.1 ± 10.2 years. Of these, 253 were allocated to the training set, while the remaining 108 formed the test set. Table 1 summarizes the demographic details and tumor characteristics for the two sets. Clinical characteristics did not differ significantly between sets, ensuring a balanced distribution. Patients were categorized into survivor (*n* = 253) and non-survivor (*n* = 108) groups based on five-year survival, with baseline characteristics detailed in Table 2. Significant differences were observed in age, sex, tumor size, histology, and pathologic stage between groups, while adjuvant therapy history showed no significant difference (*p* = 0.648).

### 3.2. Establishment of the Imaging-Based Model

**Figure 2:**
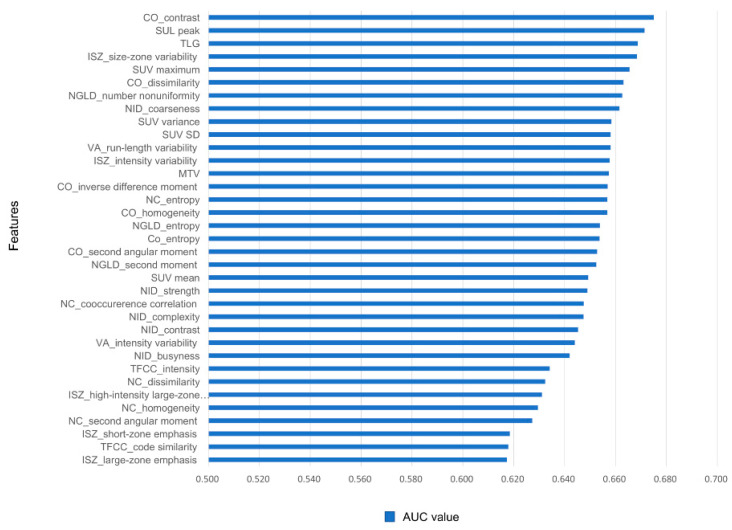
Area under the curve (AUC) values for the top 35 ^18^F-FDG PET-derived features predicting five-year survival after surgery.

**Figure 3:**
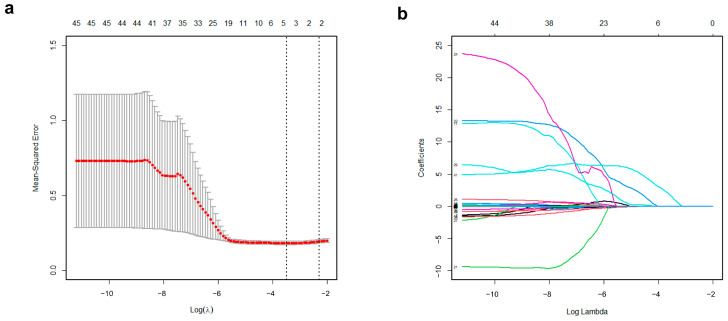
^18^F-FDG PET-derived feature selection using the least absolute shrinkage and selection operator (LASSO) logistic regression model. (**a**) Identification of the optimal penalization coefficient lambda (λ) in the LASSO model used 10-fold cross-validation and the minimum criterion. As a result, a λ value of 0.0305 was selected. (**b**) LASSO coefficient profiles of 5 features selected among 46 features. Each colored line represents the coefficient of each feature.

In the training set, the ^18^F-FDG PET-derived features were ordered by their AUC values, indicating discriminative power for the binary outcomes of the five-year survival status (Supplementary Table S2). The features with the best predictive performances were CO_contrast (0.675) and SUL peak (0.671), followed by TLG (0.669) and ISZ_size-zone variability (0.668) (Figure 2). Among the 70 features, 24 were excluded as irrelevant with poor performance (AUC < 0.6). Applying the remaining 46 features to the LASSO method, 5 were finally selected for an imaging-based risk score model (Figure 3). The predictive probability of death within five years for each patient was calculated using a simple linear combination of five selected indicators multiplied by their respective coefficients, as follows:

PET-based risk score = 0.113427981 + (0.0000002977 × CO_contrast) + (0.0000492625 × VA_intensity variability) + (0.010082504 × NID_contrast) + (0.013133648 × SUL peak) + (1.414154452 × TFCC_code similarity).

**Figure 4:**
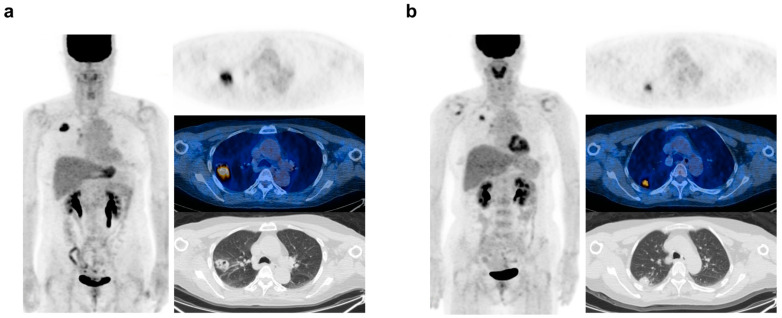
Representative PET/CT images of NSCLC patients following right upper lobectomy for adenocarcinoma. (**a**) Maximum intensity projection (left), transaxial fusion PET/CT (right middle), and CT (right bottom) images of a 74-year-old female with a high FDG-based risk score of 0.171. The patient’s pathologic tumor stage was I [T2 (tumor size 3.5 cm) N0 (no metastasis after mediastinal lymph node dissection)], but she died 20 months after surgery due to recurrence. (**b**) Maximum intensity projection (left), transaxial fusion PET/CT (right middle), and CT (right bottom) images of 61-year-old female with a low FDG-based risk score (0.076). The patient’s pathologic tumor stage was III [T2 (tumor size 2.2 cm, but invaded the visceral pleural beyond the elastic layer) N2 (identified metastasis in right pulmonary hilar and lower paratracheal lymph nodes after mediastinal lymph node dissection)], but she was alive at the five-year follow-up.

In the training set, the PET-based risk score had a median of 0.116 and an interquartile range (IQR) of 0.079–0.203. The optimal cut-off value of the risk score for predicting the five-year survival was identified as 0.111. Representative cases with high and low scores are illustrated in Figure 4.

### 3.3. Construction of Clinical and Hybrid Model

In the training set, we developed a clinical model that includes the five clinical risk factors that showed significant differences between survivor and non-survivor groups. A hybrid model was also constructed by incorporating an ^18^F-FDG PET-based risk score into the same clinical variables. The results of the two multivariable logistic regression models are shown in Table 3. In the clinical model, age (OR 1.080, 95% CI 1.041–1.120; *p* < 0.001) and tumor size (OR 1.214, 95% CI 1.018–1.448; *p* = 0.031) were identified as independent predictors of five-year survival. In the hybrid model, age (OR 1.083, 95% CI 1.043–1.125; *p* < 0.001) and the PET-based risk score (OR 5.231, 95% CI 1.987–6.932; *p* = 0.009) were identified as independent predictors of five-year survival. Tumor size (OR 1.046, 95% CI 0.848–1.291; *p* = 0.673) did not show independent significance when adjusted for the PET-based risk score.

### 3.4. Model Validtion

We applied the LASSO-based formula, developed using the training set, to the test set to calculate the PET-based risk scores for each case. The median risk score from the test set was 0.112 (IQR, 0.068–0.225), with no significant difference compared to the training set (*p* = 0.953). In the test set, the multivariable logistic regression results were consistent with the training set findings: in the clinical model, age and tumor size were independent variables, while, in the hybrid model, age and the PET-based risk score were independent predictors (Table 4).

### 3.5. Model Performance and Calibration

**Figure 5:**
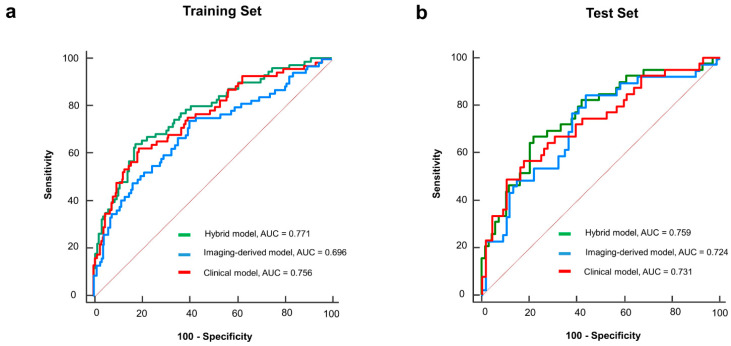
Receiver operating characteristic curves for the PET-based risk score and clinical and hybrid models in the (**a**) training set and (**b**) test set.

**Figure 6:**
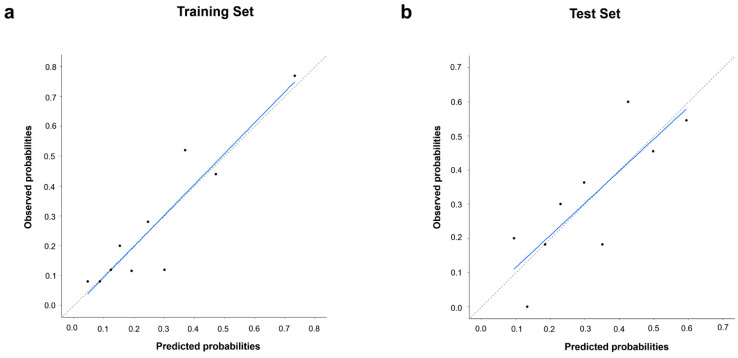
Calibration curves of hybrid models in the (**a**) training set and (**b**) test set.

In the training set, ROC curves were generated for the PET-based risk score and clinical and hybrid models (Figure 5a). The associated AUC values for the five-year survival status were 0.696 (95% CI: 0.635–0.752), 0.756 (95% CI: 0.698–0.807), and 0.771 (95% CI: 0.715–0.822), respectively. The hybrid model demonstrated the best discriminative performance for predicting the 5-year survival status. When comparing AUCs (Supplementary Table S3), the hybrid model significantly outperformed the PET-based risk score (*p* = 0.022). The performance of the hybrid model demonstrated a modest improvement over the clinical model; however, the difference was not significant (*p* = 0.233). In the test set, the hybrid model also maintained the highest AUC (0.759, 95% CI: 0.667–0.836) (Figure 5b). However, the difference was not significant compared to those of the PET-based risk score (AUC = 0.724, 95% CI: 0.630–0.806; *p* = 0.334) or the clinical model (AUC = 0.731, 95% CI: 0.637–0.811; *p* = 0.213). The calibration curve of the hybrid model with the best predictive performance revealed a good agreement between the observed outcome and prediction in the training and test sets (Figure 6). Additionally, the Hosmer–Lemeshow test yielded a non-significant statistic in two sets (*p*  =  0.360 and *p* = 0.630, respectively), indicating that the model fit well.

## 4. Discussion

In the era of precision medicine, identifying cancer patients with a poor prognosis is crucial for individualized management strategies. Biomarkers signaling a poor prognosis can improve the risk stratification beyond traditional tumor staging [27]. Recent technological advances have enabled the extraction of diverse invisible information from imaging studies in the oncological area. ^18^F-FDG PET/CT offers significant prognostic insights by reflecting the metabolic characteristics of cancer. Employing these ^18^F-FDG PET features, the current study developed an imaging-based score formula for predicting five-year survival after curative surgery in NSCLC patients. This PET-based risk score was an independent prognostic factor distinct from clinically related prognostic variables. The PET-based risk score, as demonstrated in the representative case in Figure 4, may provide prognostic information that tumor staging alone cannot capture, potentially aiding in patient management. The integrated hybrid model incorporating clinical variables demonstrated good discriminative performances for predicting survival status at five years in the training (AUC = 0.771) and testing sets (AUC = 0.759). Although the difference was not significant, the predictive performance of the hybrid model was superior to that of the clinical model alone in both the training and test sets.

Metabolic heterogeneity has recently been recognized as a crucial factor in cancer progression [28]. ^18^F-FDG PET-derived textural features can provide information more closely associated with these tumor characteristics compared to those extracted from CT [29]. In our study, among 70 PET-derived features, the contrast from the co-occurrence matrix showed the highest predictive accuracy for survival (AUC = 0.675), with other features from the same matrix also demonstrating strong performance (AUCs ranging from 0.653 to 0.663). The co-occurrence matrix represents local tumor heterogeneity by analyzing the spatial relationship between pixel intensities within an image [30]. Prior studies on PET-derived textural features in NSCLC have consistently identified those from the co-occurrence matrix as offering superior prognostic predictive power [31]. These results suggest the significance of local tumor heterogeneity over global heterogeneity in affecting patient outcomes. Additional research is required to validate these observations and investigate their implications.

Recent prognostic studies are focused on developing models that combine various associated features to improve clinical usefulness. In high-dimensional data, identifying relevant features is crucial for developing effective models, as it may include numerous unrelated variables [24]. We initially excluded irrelevant variables by analyzing the individual AUCs of the features. To apply the developed model to new clinical data, it is also necessary to reduce the risk of overfitting in the training data. Recently, the LASSO method has been widely employed in the modeling of high-dimensional data to address these issues [32]. After the LASSO method was applied, an imaging-based model that incorporates five relevant features was constructed. These features included CO_contrast and SUL_peak, each demonstrating the best individual predictive performance. However, the other three variables showed relatively lower individual predictive performance, with AUC values ranging between 0.618 and 0.645. This suggests that the LASSO model prioritizes not only high predictive accuracy but also adaptability across various datasets. In testing, the model consistently exhibited comparable predictive performance, affirming its robustness.

The Cox proportional hazards model, widely used in survival analysis, presumes that variables’ effects remain constant over time. This assumption is not always true in practice, limiting its effectiveness when hazard ratios fluctuate. Its impact could be particularly significant in the analyses of high-dimensional data containing many potential variables [18]. A recent study highlighted that a discrete-time prediction model, which estimates the survival status at a specific time point, could serve as a superior option for survival analysis with many features [19]. This alternative avoids the need for the assumption of proportional hazards. Furthermore, this binary outcome prediction method enhances the interpretability, simplifying the comprehension for both medical professionals and patients. We focused specifically on the status of survival at the five-year mark, considered a crucial time point in cancer prognosis [33]. Our model indicates that patients unlikely to survive past five years might benefit from continued therapy or close monitoring post-treatment.

Previous research has developed prognostic models using ^18^F-FDG PET-derived textural features for NSCLC. Ahn et al. introduced a random forest model based on these features to predict three-year recurrence in 93 stage I–III NSCLC patients post-curative surgery, achieving an AUC of 0.956 [34]. Another study utilized a naive Bayes model to forecast the two-year recurrence in 77 stage I–III NSCLC patients treated with curative intent, reaching an AUC of 0.816 [35]. However, for the accurate analysis of high-dimensional data, machine learning methods typically require larger sample sizes. The LASSO technique is effective for high-dimensional, low-sample-size data analysis [32]. Li et al. developed a model using pretreatment ^18^F-FDG PET/CT textural features and the LASSO method in 368 NSCLC patients, demonstrating its utility in overall survival prediction. This model, combining clinical and imaging-based scores, outperformed a clinical-only model in OS prediction, with an AUC of 0.891 versus 0.846 [16]. Yang et al. found that adding PET/CT-based risk scores to clinical parameters significantly improved the OS prediction in 315 NSCLC patients, showing high concordance index values in both the training (0.776) and validation (0.789) cohorts [17]. Our study also showed a modest improvement in the predictive performance for survival outcomes using a hybrid model that integrates clinical variables and PET-based risk scores relative to using a clinical-only model. However, distinct from prior studies that comprised a broad range of patient populations (stages I–IV) undergoing both curative and palliative treatments, our research focused exclusively on a larger cohort receiving curative therapy. Our model can provide more valuable insights for post-surgery follow-up strategies or decisions on adjuvant therapy.

PET-based feature analysis has several remaining challenges. A major issue is textural feature variation due to differing tumor delineation methods [36]. Advanced tumor segmentation techniques that apply deep learning for improved tumor delineation have been introduced but not validated [37]. Our study used PET Edge, a validated, widely-used, gradient-based delineation method [20]. This semi-automated method improves feature value reproducibility by reducing inter-observer variability, surpassing manual segmentation. Moreover, it can better capture necrotic portions associated with prognosis compared to models with fixed thresholds [38]. Variability due to the use of different software is also a major issue. Recently, the International Biomarker Standardization Initiative (IBSI) recommended using LifeX or pyradiomics in radiomics feature analysis to address this. These features are advantageous for comparing radiomics features across multi-imaging modalities because they are commonly used in PET, CT, and MRI [39]. However, the CGITA software we used is focused on PET texture analysis, and its clinical utility has been validated [21]. Future studies are warranted to compare the prognostic significance of ^18^F-FDG PET-derived textural features extracted using various software tools.

Our study has several limitations. First, its retrospective design and the use of single-center data introduce inherent biases and limit the generalizability of our findings. Additionally, the limited sample size affects the power of our conclusions. The predictive model was not externally validated with an independent cohort from another institution, which may further restrict its generalizability and robustness. Potential confounding variables that were not considered also could have influenced the results. Last, certain cases had a short follow-up period, which might impact the assessment of long-term survival outcomes.

## 5. Conclusions

Among the PET-derived features, CO_contrast and SUL_peak demonstrated the best individual predictive performance for five-year survival status and were included in the LASSO-based formula. The ^18^F-FDG PET-based risk score, derived using the LASSO method, served as an independent prognostic indicator, distinct from clinical variables, for predicting the five-year survival status in patients with NSCLC who underwent curative surgery. Our PET-based risk score, particularly when combined with clinical variables, offers a valuable tool for risk stratification in NSCLC following curative surgery. This model can aid clinicians in identifying high-risk patients who may benefit from more aggressive follow-up and adjuvant therapies, ultimately improving personalized treatment strategies and patient outcomes. Future research should focus on validating our model with independent, multi-center cohorts to enhance the generalizability. Additionally, by comparing the prognostic value of the PET-derived features using different image analysis software and exploring advanced tumor segmentation techniques.

## Figures and Tables

**Table 1:** Clinical characteristics of the training set and the test set.

Variable	Total (*n* = 361)	Training Set (*n* = 253)	Test Set (*n* = 108)	*p* Value
Age, years (mean ± SD)	63.1 ± 10.2	62.8 ± 10.2	63.8 ± 10.2	0.382
Sex, male (%)	237 (65.7)	161 (63.6)	76 (70.3)	0.218
Tumor size, cm (mean ± SD)	3.6 ± 1.9	3.7 ± 2.0	3.5 ± 1.6	0.327
Histology				0.762
Adenocarcinoma (%)	232 (64.3)	165 (65.2)	67 (62.0)	
Squamous cell carcinoma (%)	104 (28.8)	70 (27.7)	34 (31.5)	
Others (%)	25 (6.9)	18 (7.1)	7 (6.5)	
Tumor stage				0.526
I (%)	140 (38.8)	95 (37.6)	45 (41.7)	
II (%)	105 (29.1)	78 (30.8)	27 (25.0)	
III (%)	116 (32.1)	80 (31.6)	36 (33.3)	
Type of surgery				0.438
Pneumonectomy (%)	12 (3.3)	6 (2.4)	6 (5.6)	
Bilobectomy (%)	17 (4.7)	11 (4.4)	6 (5.6)	
Lobectomy (%)	307 (85.1)	218 (86.1)	89 (82.4)	
Segmentectomy (%)	25 (6.9)	18 (7.1)	7 (6.4)	
Adjuvant therapy				0.466
None (%)	204 (56.5)	138 (54.5)	66 (61.1)	
Chemotherapy (%)	128 (35.5)	94 (37.2)	34 (31.5)	
Radiotherapy (%)	3 (0.8)	3 (1.2)	0 (0.0)	
Chemoradiotherapy (%)	26 (7.2)	18 (7.1)	8 (7.4)	
Death within five years (%)	108 (29.9)	69 (27.3)	39 (36.1)	0.094

Data are presented as numbers (%) or mean ± standard deviation.

**Table 2:** Comparison of clinical characteristics between survivor and non-survivor groups.

Variable	Non-Survivor (*n* = 108)	Survivor (*n* = 253)	*p* Value
Age, years (mean ± SD)	67.6 ± 9.3	61.2 ± 10.0	<0.001
Sex, male (%)	83 (76.9)	154 (60.9)	0.004
Tumor size, cm (mean ± SD)	4.5 ± 2.4	3.3 ± 1.5	<0.001
Histology, ADC (%)	56 (51.9)	176 (69.6)	0.001
Tumor stage, II/III (%)	80 (74.1)	141 (55.7)	0.001
Adjuvant therapy, presence (%)	45 (41.7)	112 (44.3)	0.648

Data are presented as numbers (%) or mean ± standard deviation. SD, standard deviation; ADC, adenocarcinoma.

**Table 3:** Multivariable logistic regression analysis of the training set.

Variables	Clinical Model			Hybrid Model		
	Adjusted OR	95% CI	*p* Value	Adjusted OR	95% CI	*p* Value
Age, years	1.080	1.041–1.120	<0.001	1.083	1.043–1.125	<0.001
Sex (M vs. F)	1.337	0.644–2.772	0.436	1.038	0.486–2.217	0.924
Tumor stage (III/II vs. I)	1.567	0.733–3.351	0.247	1.301	0.595–2.845	0.510
Histology (ADC vs. others)	0.667	0.334–1.335	0.253	0.881	0.421–1.845	0.737
Tumor size, cm	1.214	1.018–1.448	0.031	1.046	0.848–1.291	0.673
PET-based risk score				5.231	1.987–6.932	0.009

OR, odds ratio; CI, confidence interval; ADC, adenocarcinoma.

**Table 4:** Multivariable logistic regression analysis of the test set.

Variables	Clinical Model			Hybrid Model		
	Adjusted OR	95% CI	*p* Value	Adjusted OR	95% CI	*p* Value
Age, years	1.051	1.003–1.100	0.036	1.060	1.010–1.113	0.019
Sex (M vs. F)	1.538	0.548–4.316	0.414	1.450	0.500–4.201	0.494
Tumor stage (III/II vs. I)	1.392	0.480–4.041	0.543	1.386	0.462–4.156	0.560
Histology (ADC vs. others)	1.323	0.506–3.456	0.568	1.695	0.614–4.680	0.309
Tumor size, cm	1.475	1.046–2.080	0.027	1.008	0.619–1.642	0.976
PET based-risk score				1311.521	1.901–904,972.132	0.031

HR, hazard ratio; CI, confidence interval; ADC, adenocarcinoma.

## Data Availability

Restrictions apply to the availability of these data. Data were obtained from the Samsung Medical Center and are available from the corresponding author with the permission of the Samsung Medical Center.

## Supplementary Materials

The following supporting information can be downloaded at: https://www.mdpi.com/article/10.3390/cancers16142525/s1, Supplementary Table S1: List of 70 quantitative PET-based radiomic features. Supplementary Table S2: AUC values of 46 relevant ^18^F-FDG PET-based features. Supplementary Table S3: Comparison of predictive performances in three models.
