# Supplementary material for: 18F-Fluorodeoxyglucose Positron Emission Tomography-Based Risk Score Model for Prediction of Five-Year Survival Outcome after Curative Resection of Non-Small-Cell Lung Cancer
Source: Cancers (Basel). 2024 Jul 12;16(14):2525. doi: 10.3390/cancers16142525 (PMC11274931; doi:10.3390/cancers16142525)
Supplement: Supplementary file 1 [file cancers-16-02525-s001.zip › cancers-3058735-supplementary.pdf]

^{18}F-FDG PET-based Risk Score Model for Prediction of 5-year Survival Outcome after Curative Resection of Non-Small Cell Lung Cancer

Supplementary Table S1. List of 70 Quantitative PET-Based Radiomic Features

Parent matrix and abbreviation	Radiomic features
Co-occurrence (CO)	Second angular moment, contrast, entropy, homogeneity, dissimilarity, inverse difference moment
Voxel-alignment (VA)	Short-run emphasis, long-run emphasis, intensity variability, run-length variability, run percentage, low-intensity run emphasis, high-intensity run emphasis, low-intensity short-run emphasis, high-intensity short-run emphasis, low-intensity long-run emphasis, high-intensity long-run emphasis
Neighborhood intensity difference (NID)	Coarseness, contrast, busyness, complexity, strength
Intensity size-zone (ISZ)	Short-zone emphasis, large-zone emphasis, intensity variability, size-zone variability, zone percentage, low-intensity zone emphasis, high-intensity zone emphasis, low-intensity short-zone emphasis, high-intensity short-zone emphasis, low-intensity large-zone emphasis, high-intensity large-zone emphasis
Normalized co-occurrence (NC)	Second angular moment, contrast, entropy, homogeneity, inverse difference moment, dissimilarity, cooccurrence correlation
Voxel statics	Minimum SUV, maximum SUV, mean SUV, SUV variance, SUV SD, SUV skewness, SUV kurtosis, TLG, tumor volume, entropy, SULpeak
Texture spectrum (TS)	Max spectrum, Black-white symmetry
Texture feature coding (TFC)	Coarseness, homogeneity, mean convergence, variance
Texture feature coding cooccurrence matrix (TFCC)	Second angular moment, contrast, entropy, homogeneity, intensity, inverse difference moment, code entropy, code similarity
Neighborhood gray-level dependence (NGLD)	Small-number emphasis, large-number emphasis, number nonuniformity, second moment, entropy

SUV, standardized uptake value; SD, standard deviation; TLG, total lesion glycolysis

Supplementary Table S2. AUC values of relevant 46 ^{18}F-FDG PET-based features

Features	AUC values
CO contrast	0.6747795
SUL peak	0.6713926
TLG	0.6687933
ISZ size-zone variability	0.6684389
SUV maximum	0.6654852
CO dissimilarity	0.6631222
NGLD number nonuniformity	0.6626497
NID coarseness	0.6615469
SUV variance	0.6583963
VA run-length variability	0.6580813
SUV SD	0.6580813
ISZ intensity variability	0.6576087
MTV	0.6573724
CO inverse difference moment	0.6568998
CO homogeneity	0.6568210
NC entropy	0.6568210
NGLD entropy	0.6537886
CO entropy	0.6537098
CO second angular moment	0.6527647
NGLD second moment	0.6524496
SUV mean	0.6492596
NID strength	0.6489445
NC co-occurrence correlation	0.6475268
NID complexity	0.6474480
NID contrast	0.6452426
VA intensity variability	0.6439824
NID busyness	0.6420132
TFCC intensity	0.6341367
NC dissimilarity	0.6323251
ISZ high-intensity large-zone emphasis	0.6310649
NC homogeneity	0.6294896
NC second angular moment	0.6272842
ISZ short-zone emphasis	0.6183837
TFCC code similarity	0.6178324
ISZ large-zone emphasis	0.6173204
VA short-run emphasis	0.6172023
VA long-run emphasis	0.6167297
ISZ large-zone emphasis	0.6165721
NC inverse difference moment	0.6148393
NGLD large-number emphasis	0.6114130
NC contrast	0.6105072
ISZ zone percentage	0.6103103
SUV entropy	0.6099559
SUV kurtosis	0.6034972
NGLD small number emphasis	0.6023551
ISZ low-intensity short-zone emphasis	0.6020794

Supplementary Table S3. Comparison of predictive performances in three models.

	AUC (95% CI)	*P* value*	*P* value†	*P* value‡
Training set				
PET-based risk score model	0.696 (0.635 – 0.752)	-	0.130	0.022
Clinical model	0.756 (0.698 – 0.807)	0.130	-	0.233
Hybrid model	0.771 (0.715 - 0.822)	0.022	0.233	-
Test set				
PET-based risk score model	0.724 (0.630-0.806)	-	0.890	0.334
Clinical model	0.731 (0.637-0.811)	0.890	-	0.213
Hybrid model	0.759 (0.667-0.836)	0.334	0.213	-

*Comparison with the AUC of the PET-based risk score model.
†Comparison with the AUC of the clinical model.
‡Comparison with the AUC of the hybrid model.
